# American Institute of Homœopathy

**Published:** 1894-07

**Authors:** 


The American Institute of Homœopathy held its Jubilee session at Denver, in June. The proceedings of the meeting were very interesting and very voluminous. Unfortunately, the space at our disposal in this number will not allow of even a condensed report. An attempt will be made in the August number to give a general idea of the work done.

